# Development and validation of a nomogram for predicting the risk of threatened abortion after in vitro fertilization-embryo transfer

**DOI:** 10.12669/pjms.42.2.13419

**Published:** 2026-02

**Authors:** Jia Yu, Xudong Zhang, Lihua Xia

**Affiliations:** 1 Jia Yu Department of Reproductive and Fetal Care Ward, Huzhou Maternity & Child Health Care Hospital, Huzhou, Zhejiang Province 313000, P.R. China; 2 Xudong Zhang Department of Reproductive and Fetal Care Ward, Huzhou Maternity & Child Health Care Hospital, Huzhou, Zhejiang Province 313000, P.R. China; 3 Lihua Xia Department of Reproductive and Fetal Care Ward, Huzhou Maternity & Child Health Care Hospital, Huzhou, Zhejiang Province 313000, P.R. China

**Keywords:** Embryo transfer, Nomogram, In Vitro Fertilization, Threatened abortion

## Abstract

**Objective:**

This study aimed to develop and validate a nomogram for predicting the risk of threatened abortion after in vitro fertilization-embryo transfer (IVF-ET).

**Methodology:**

Clinical records of 409 patients who underwent IVF-ET treatment due to tubal factors in Huzhou Maternity & Child Health Care Hospital from January 2017 to May 2025 were retrospectively selected. Patients were randomly assigned to the training (n=286) and validation (n=123) cohorts in a 7:3 ratio. The Least Absolute Shrinkage and Selection Operator (LASSO) method and multivariate logistic regression were applied to identify independent risk factors, which were then used to construct a nomogram for predicting the risk of threatened abortion. The nomogram was validated using the area under the receiver operating characteristic (ROC) curve (AUC) and decision curve analysis (DCA) to assess its clinical application value.

**Results:**

Female age, endometrial thickness, number of embryos transferred, and progesterone (P) level 14 days after IVF-ET were identified as risk factors for threatened abortion (P<0.05). Based on the four independent factors, a nomogram was developed. The nomogram demonstrated sufficient predictive accuracy, with AUC values of 0.822 (95% confidence interval (CI): 0.737-0.907) and 0.822 (95% CI: 0.724-0.919) in the training and validation cohorts, respectively. The validation results showed that the consistency index (C-index) for the training and validation cohorts was 0.802 (95% CI: 0.715-0.889) and 0.807 (95% CI: 0.719-0.895), respectively. The calibration curves for the two cohorts are closer to the diagonal (the ideal curve).

**Conclusions:**

The established nomogram for threatened abortion after IVF-ET has good predictive value and helps identify high-risk populations.

## INTRODUCTION

In recent years, the incidence of infertility worldwide has been on the rise due to various factors.^1^,^2^ In vitro fertilization and embryo transfer (IVF-ET) technology has become one of the primary means to solve infertility problems.^3^ However, although the pregnancy rate after IVF-ET can reach up to 60%, some patients still experience threatened abortion, which seriously affects pregnancy outcomes.^3^,^4^ According to epidemiological investigations, the incidence of threatened abortion after IVF-ET is about 15% -30%, significantly higher than the incidence of threatened abortion in natural pregnancy (about 10% -15%).^5^,^6^

The risk factors of threatened abortion after IVF-ET include advanced age, insufficient endometrial thickness, abnormal morphology, intrauterine adhesions, luteal insufficiency, thyroid dysfunction, and polycystic ovary syndrome.^7^-^10^ In addition, unhealthy lifestyle habits, pre-thrombotic state, and multiple transplant history have also been confirmed to be closely related to threatened abortion after IVF-ET.^11^,^12^ However, there is still no validated method to incorporate these risk factors into a nomogram predictive model that may allow clinicians to timely assess the risk of spontaneous abortion.^13^ This study aimed to construct and validate a nomogram prediction model to guide the management of subsequent pregnancies.

## METHODOLOGY

This study retrospectively analyzed patients who underwent IVF-ET treatment for tubal factors at Huzhou Maternity & Child Health Care Hospital from January 2017 to May 2025. The clinical data of all 409 eligible patients were extracted from the hospital’s electronic medical record (EMR) system in a single batch in June 2025 using standardized query procedures to ensure consistency. All patients who met the inclusion and exclusion criteria were enrolled consecutively to minimize selection bias. Threatened abortion in this study was defined as occurring strictly within the first trimester, described as a gestational age of 12 weeks or less, in accordance with established clinical guidelines. Patients were randomly allocated to the training and validation cohorts in a 7:3 ratio using a computer-generated randomization process implemented in R software (version 4.2.2), with a fixed random seed to ensure reproducibility.

### Ethical Approval:

The hospital’s ethics committee approved the study under number 2025-J-059, Date: August 17^th^, 2025. All patients included in this retrospective study had provided written informed consent at the time of IVF-ET treatment for the use of their clinical data in research. In addition, all data extracted from the hospital’s electronic medical records were fully anonymized prior to analysis to ensure patient privacy and compliance with ethical standards.

### Inclusion criteria:

- Underwent IVF-ET treatment.
- Confirmed clinical intrauterine pregnancy, defined as the presence of a gestational sac on ultrasound examination.
- Diagnosed with early threatened abortion within the first trimester (gestational age ≤12 weeks), presenting with symptoms such as vaginal bleeding and/or lower abdominal pain, with or without accompanying ultrasound findings.
- Complete medical records.

### Exclusion criteria:

- Ectopic pregnancy.
- Definitive spontaneous abortion or induced abortion occurring before clinical data collection and endpoint definition.
- Uterine malformation, uterine mucosal fibroids, and cervical adhesions.
- Chromosomal abnormalities and genetic diseases.
- Significant endocrine disorders, hypertension, diabetes mellitus, anemia, or active infections, including Toxoplasma gondii and cytomegalovirus.

### Data collection and variable definition:

Clinical data of all pregnant women, including age, body mass index (BMI), parity, history of ectopic pregnancy, anti-Mullerian hormone (AMH) level, number of transplants, total number of transplantable embryos, number of transplanted embryos, endometrial thickness, estradiol (E2) and progesterone (P) level two days after oocyte retrieval, beta human chorionic gonadotropin (beta-hCG), E2 and P level 14 days after transplantation were collected.

The number of transplants in this study referred to the cumulative number of embryo transfer procedures performed on a patient during the study period, including both fresh embryo transfer and frozen-thawed embryo transfer cycles. A value of two or more indicated that the patient underwent multiple IVF-ET cycles. The number of transplantable embryos refers to embryos created in vitro following fertilization and assessed to be of transferable quality. The number of embryos transferred refers to the actual number of embryos placed into the uterine cavity in a single embryo transfer procedure. In this study, this variable was categorized into one versus two embryos transferred.

### Statistical analysis:

All patients were randomly assigned to the training and validation cohorts in a 7:3 ratio. Statistical analyses were performed using SPSS version 27.0 (IBM Corp, NY, USA). The Shapiro-Wilk test was used to evaluate the normality of the evaluation data. Normal distribution data were represented by mean ± standard deviation and subjected to an independent sample t-test. Non-normally distributed data were described by median and interquartile range, and subjected to the Mann-Whitney U test. A chi-square test was performed on count data. The primary endpoint of this study was the diagnosis of threatened abortion.

Variable selection was performed using the Least Absolute Shrinkage and Selection Operator (LASSO) regression with 10-fold cross-validation. The optimal penalty parameter λ was chosen using the one-standard-error (1-SE) rule, which selects the most parsimonious model within one standard error of the minimum cross-validation error. Variables with non-zero coefficients were considered potential predictors of threatened abortion after IVF-ET. These variables were subsequently entered into a multivariate logistic regression model using a backward stepwise selection method, with entry and removal criteria set at p < 0.10 and p > 0.15, respectively. Before multivariate analysis, multicollinearity was assessed using the variance inflation factor (VIF), and all VIFs were below 2.0, indicating no significant multicollinearity. The nomogram was developed based on the final logistic regression model. Statistical analysis and figure generation were conducted using SPSS version 27.0 (IBM Corp., NY, USA) and R version 4.2.2 (R Foundation for Statistical Computing, Vienna, Austria), using the rms, pROC, and Decision Curve Analysis (DCA) packages. Internal validation of the model was conducted using 1000 bootstrap resamples to calculate the optimism-corrected concordance index (C-index), providing a robust estimate of model discrimination. Calibration curves were generated for both the training and validation cohorts, and ROC analysis was used to evaluate discriminative ability based on the area under the curve (AUC). Decision curve analysis was performed to assess the net clinical benefit across a range of risk thresholds.

In multivariate analysis, p-values were based on the Wald test. A P-value<0.05 was considered significant. Scores were assigned to each value level and each influencing factor, and the values of each variable were mapped to a scale of 0 to 100. The length of the line segment reflected the contribution of the factor to the target event. The predicted probability of threatened abortion was obtained through the functional transformation relationship between the total score and the probability of threatened abortion. The validation cohort was used to evaluate the predictive ability of the developed nomogram, including differentiation and calibration. Calibration curves were generated to illustrate potential differences between the training and validation cohorts, including the original and recalibrated nomograms.

The model’s discriminative ability was evaluated using receiver operating characteristic (ROC) curve analysis, with the area under the curve (AUC) as the metric. The predictive ability of the final model was evaluated by comparing the observed rates of threatened abortion. In addition, decision curve analysis (DCA) was conducted to assess the clinical value of the model and to calculate net benefits at various risk threshold probabilities, thereby determining its real-world application.

## RESULTS

This study included 409 eligible patients who underwent IVF-ET treatment due to tubal factors. The age range of patients was 23-43 years, with a median of 30 (27, 33) years. During pregnancy, 59 patients were diagnosed with threatened abortion, with an incidence of 14.4% (59/409). All patients were randomly divided into a training cohort (n=286) and a validation cohort (n=123) in a 7:3 ratio. As shown in Table-I, there was no significant difference in the incidence rates between the training cohort and the validation cohort (12.9% (37/286) and 17.9% (22/123), respectively) (P>0.05). Clinical characteristics of the training and the validation cohorts were similar (P>0.05).

**Table-I:** Clinical Characteristics of IVF-ET Patients.

Variables	Training cohort (n=286)	Validation cohort (n=123)	Z/t/χ^2^	P
Age (years)			0.921	0.337
<35	237 (82.9)	97 (78.9)		
≥35	49 (17.1)	26 (21.1)		
Female BMI (kg/m^2^)	22.33 (20.3, 24.7)	21.6 (20.2, 23.9)	-1.078	0.281
Number of pregnancies	1 (0, 2)	1 (0, 2)	-1.517	0.129
Multiparous women (yes)	54 (18.9)	25 (20.3)	0.115	0.734
History of ectopic pregnancy (yes)	85 (29.7)	45 (36.6)	1.870	0.172
AMH (ng/ml)	3.73 (2.47, 5.43)	3.42 (2.45, 5.48)	-0.279	0.781
Number of transplants			0.063	0.802
1	263 (92.0)	114 (92.7)		
≥2	23 (8.0)	9 (7.3)		
The number of transplantable embryos	3 (2, 5)	3 (2, 5)	-0.704	0.481
Number of embryos transferred			0.014	0.905
1	140 (49.0)	61 (49.6)		
2	146 (51.0)	65 (50.4)		
Endometrial thickness (mm)	10 (9, 11)	10 (9, 12)	-1.520	0.128
E2 level two days after oocyte retrieval (pmol/L)	4614(3117, 6541)	4662 (2964, 6430)	-0.442	0.658
P level two days after oocyte retrieval (nmol/L)	126.5 (98.9, 186.1)	121.6 (98.9, 184)	-0.695	0.487
Beta-hCG level 14 days after IVF-ET (mIU/mL)	617.85 (302.4, 1241)	741 (351.6, 1311)	-1.324	0.186
E2 level 14 days after IVF-ET (pmol/L)	1884 (1467, 2641)	2133 (1358, 2641)	-0.171	0.864
P level 14 days after IVF-ET (nmol/L)	109 (78, 125)	109 (78, 121)	-0.373	0.709
threatened abortion (yes)	37 (12.9)	22(17.9)	1.707	0.191

***Note:*** IVF-ET, in vitro fertilization-embryo transfer; BMI, body mass index; AMH, anti - Mullerian hormone; E2, estradiol; P, progesterone; Beta-hCG, beta-human chorionic gonadotropin.

**Fig.1:**
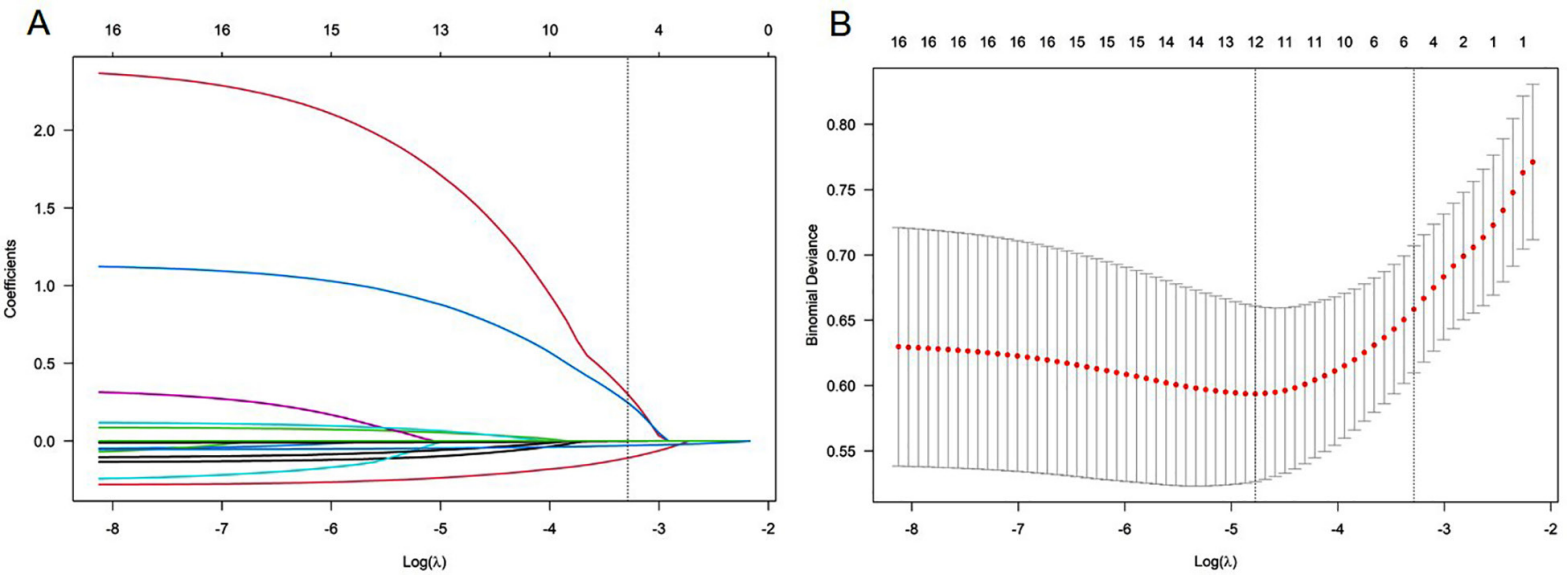
LASSO coefficient curve of threatened abortion. **A.** Each curve in the graph represents the coefficient variation of each variable. The vertical axis represents the coefficient values, the lower horizontal axis represents log (λ), and the upper horizontal axis represents the number of non-zero coefficients in the model at this time. **B.** 10-fold cross-validation fitting, then select the model.

The LASSO regression algorithm was used for feature selection to screen and construct model variables in the training cohort. LASSO regression retained five non-zero coefficient variables (Fig.1). These variables were considered significantly associated with the risk of threatened abortion. The determined variables included female age, endometrial thickness, number of transplanted embryos, Beta-hCG level 14 days after IVF-ET, and P level 14 days after IVF-ET.

To further investigate their predictive significance, a logistic regression analysis was performed on the five variables selected by LASSO. The results showed that all four variables were independent influencing factors for threatened abortion, including female age ≥ 35 years (odds ratio (OR)=3.634; 95% confidence interval (CI)=1.447-9.128; P=0.006), endometrial thickness (OR=0.756; 95% CI=0.603-0.947; P=0.015), number of transplanted embryos (OR=2.502; 95% CI=1.054-5.938; P=0.038), and P level 14 days after IVF-ET (OR=0.953; 95% CI=0.935-0.971; P<0.001) are significant predictive factors for threatened abortion after IVF-ET. The detailed results of logistic regression analysis are shown in Table-II.

**Table-II:** Analysis of Risk Factors for threatened abortion.

Independent variables	B	95% CI	P
Female age ≥ 35 years	1.290	3.634 (1.447-9.128)	0.006
Endometrial thickness	-0.280	0.756 (0.603-0.947)	0.015
Number of embryos transferred (two embryos)	0.917	2.502 (1.054-5.938)	0.038
P level 14 days after IVF-ET	-0.048	0.953 (0.935-0.971)	<0.001

***Note:*** IVF-ET, in vitro fertilization-embryo transfer; P, progesterone.

**Fig.2:**
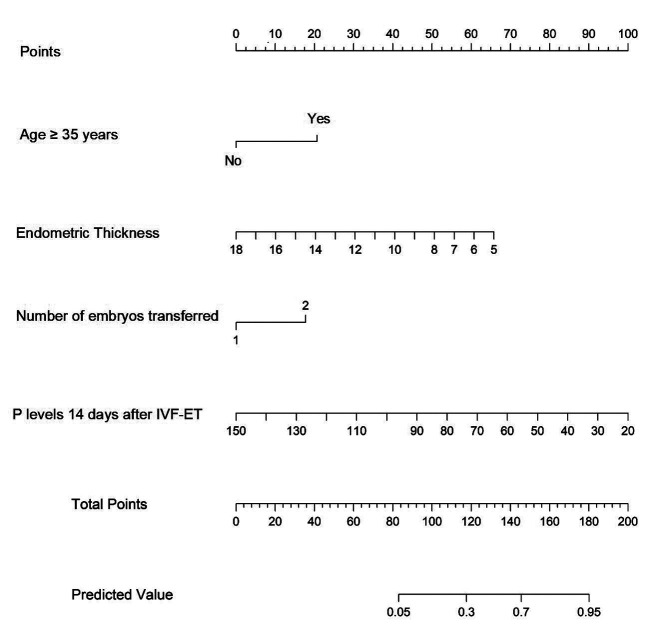
Nomogram of the risk prediction model for threatened abortion. Each level of the predictor variable represents a specific score. The total score is generated by summarizing the scores of each predictor variable. The total score corresponds to the probability of threatened abortion.

A nomogram for predicting the risk of threatened abortion after IVF-ET was constructed based on the four independent risk factors mentioned above (Fig.2). Based on the scores corresponding to each predictive indicator in the nomogram, the sum of these score values is recorded as the total score. The predicted probability corresponding to the total score represents the risk of threatened abortion. Based on the individual patient characteristics, the corresponding scores for each variable can be obtained by projecting onto the top “point” axis. Similarly, the total score is obtained by summing the scores for each variable. By projecting the total score onto the “risk of threatened abortion” axis, the probability of threatened abortion after IVF-ET can be estimated. For example, a 38-year-old pregnant woman (20 points) with an endometrial thickness of 10 mm (40 points) and two embryos transferred (18 points) had a P level of 60nmol/L (70 points) 14 days after IVF-ET, for a total of 148 points. This means that the probability of predicting threatened abortion is approximately 68%.

**Fig.3:**
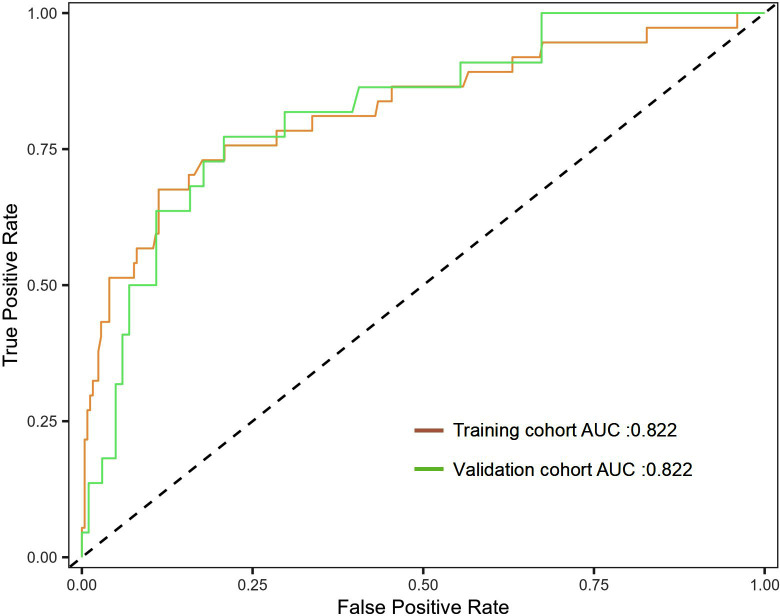
ROC curve and AUC of the nomogram. ROC: receiver operating characteristic; AUC: area under the curve.

**Fig.4:**
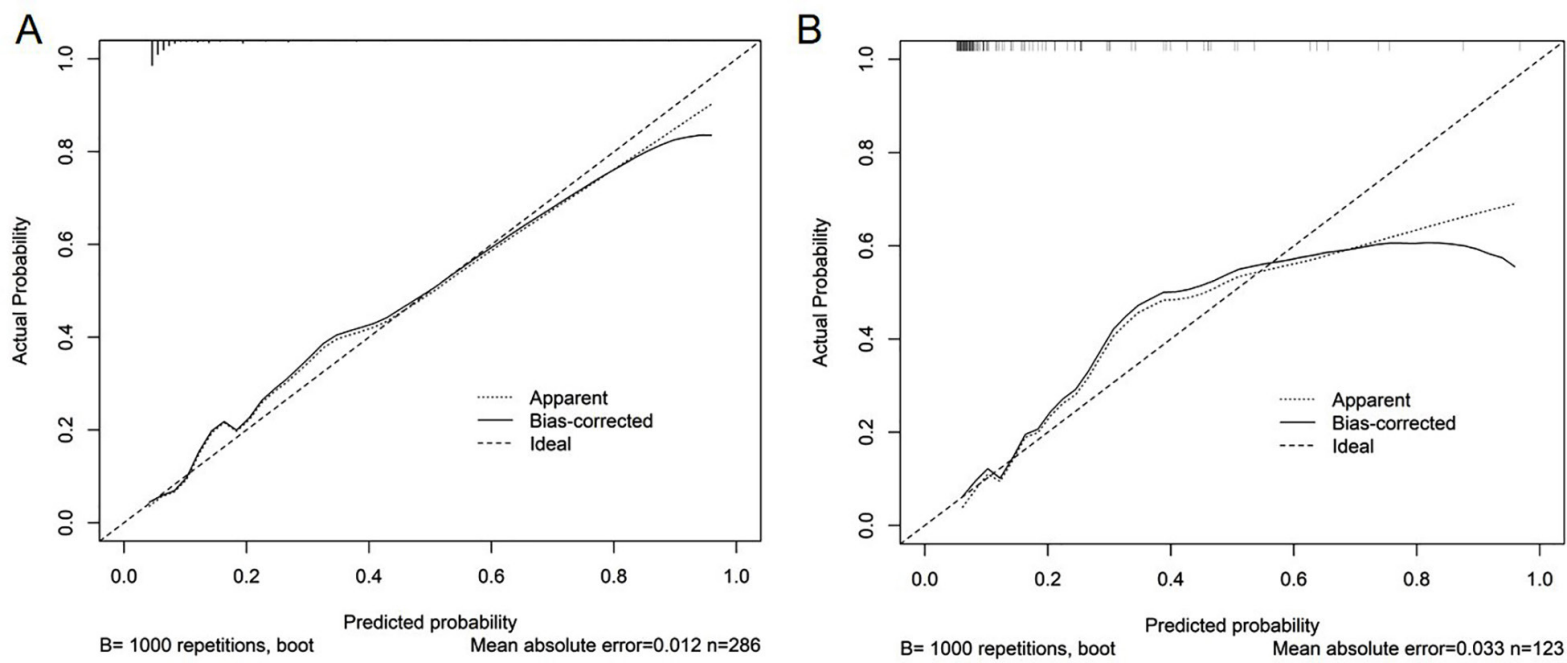
Calibration chart of the prediction model. A. Calibration chart of the training cohort. B. Calibration chart in the internal validation cohort. The x-axis represents the predicted probability of threatened abortion after IVF-ET. The y-axis represents the observed threatened abortion. The diagonal dashed line represents the perfect prediction of the ideal model. The solid line represents the performance of the nomogram. It indicates that solid lines are closer to diagonal dashed lines for better prediction.

**Fig.5:**
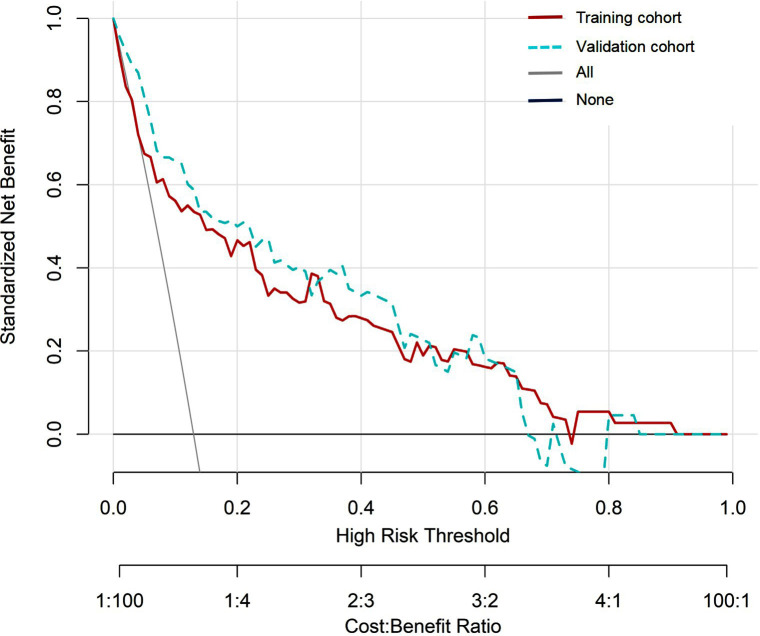
DCA of the nomogram. The x-axis displays the threshold probability, while the y-axis measures the net benefit calculated by adding true positives and subtracting false positives. DCA: decision curve analysis.

The Hosmer-Lemeshow test showed χ^2^=7.694, P=0.464 in the training cohort, and χ^2^=11.433, P=0.121 in the validation cohort, indicating that the predicted results are close to the observed results. The ROC curve in the training cohort showed good discriminability (AUC: 0.822, 95%CI: 0.737-0.907, sensitivity=29.7%, specificity=98.4%, positive predictive value (PPV) = 73.3%, negative predictive value (NPV) = 90.4%), and the C-index of bootstrap validation (1000 bootstrap samples) was 0.802 (95%CI: 0.715-0.889), indicating good predictive performance. The discriminative performance of the model was validated in the validation cohort (AUC: 0.822; 95% CI: 0.724-0.919, sensitivity=13.6%, specificity=98.0%, (PPV) = 60.0%, (NPV) = 83.9%) (Fig.3), with a C-index of 0.807 (95% CI: 0.719-0.895). In addition, calibration curve analysis showed good consistency between predicted probabilities and observed rates of threatened abortion in both the training cohort and validation cohort (Fig.4). DCA curves were drawn using training cohort data and validation cohort data separately (Fig.5). The DCA curves of the training cohort and validation cohort indicated that the prediction model has good clinical practicality.

## DISCUSSION

This retrospective cohort study developed a nomogram using a training cohort consisting of 286 patients. The nomogram was then validated to predict the occurrence of threatened abortion after IVF-ET using a validation cohort consisting of 123 patients. The developed nomogram has a good predictive value and may help identify high-risk populations.

In this study, the incidence of threatened abortion after IVF-ET was 14.4% (59/409), which is higher than the report by Huang et al.^14^ However, Valladares Garrido et al.^15^ reported that 14.7% of 218 pregnant women in Peru experienced threatened abortion during pregnancy, which is consistent with the results of this study. Notably, this study only included patients who were infertile due to tubal factors. Usually, the incidence of threatened abortion is higher in this group of patients due to the influence of tubal lesions.^14^-^16^ Furthermore, this study had a small sample size, and some patients with infertility due to uterine malformations, uterine mucosal fibroids, and cervical adhesions were excluded. In addition, the age range of the population in this study was 23-43 years, with a median age of 30 years and relatively good fertility.^17^,^18^

Threatened abortion can cause significant physical and psychological distress to pregnant women, especially in women receiving IVF-ET treatment.^17^-^19^ At present, there is no effective treatment or prevention strategy to control the occurrence of threatened abortion.^14^-^19^ Although some medications, including progesterone, are often prescribed in clinical practice, half of threatened abortions eventually develop into miscarriage.^18^-^21^ Therefore, identifying the population with a higher risk of threatened abortion is crucial.

This study developed a simple and effective nomogram to predict the likelihood of threatened abortion after IVF-ET. The prediction model includes age (≥ 35 years), number of transplanted embryos, endometrial thickness, and P level on the 14th day of transplantation. The research results show that the probability of threatened abortion increases by 3.634 times (95% CI: 1.447-9.128) for women who reach the age of 35. This is consistent with previous research findings.^18^,^22^

The results of this study are also consistent with previous reports that have demonstrated that advanced maternal age (>35) is associated with an increased rate of chromosomal abnormalities in eggs, a decline in endocrine function, and changes in the uterine environment, leading to a significant decrease in the “tolerance rate” of embryo implantation and development, and an increased risk of threatened abortion.^21^,^22^ Therefore, optimizing physical condition, strengthening monitoring and intervention during pregnancy, and targeted prevention of complications before IVF-ET can significantly reduce risks and improve pregnancy outcomes.^18^-^22^

Consistent with previous studies,^23^,^24^ endometrial thickness was negatively correlated with threatened abortion. A thin endometrium (due to multiple uterine procedures, infections, insufficient estrogen levels, etc.) has sparse vascular distribution, making it difficult to form a stable placental circulation, and increasing the risk of embryonal developmental arrest and subsequent abortion due to lack of blood supply and nutrition.^24^,^25^

The association of the number of transplanted embryos with the risk of threatened abortion in this study is consistent with previous reports. Transferring two embryos significantly increases the probability of a twin pregnancy, which is associated with greater physiological demands.^6^,^26^ Twin pregnancies can lead to excessive uterine distension, heightened hormonal fluctuations, increased placental complications, and higher maternal metabolic burden, all of which may contribute to an elevated risk of threatened abortion.^6^,^8^,^14^,^26^ Additionally, patients undergoing multiple embryo transfers may have experienced prior implantation failures, which could suggest underlying endometrial receptivity impairment or immunological dysfunction, further increasing the risk of miscarriage.^8^,^19^

The number of transplants reflects the patient’s IVF-ET treatment process. The results of this study showed that the risk of threatened abortion increased by 2.502 times (95% CI: 1.054–5.938) when two embryos were transferred, compared with single-embryo transfer. Balen et al.^26^ found that the number of lost IVF-ET twin pregnancies is significantly higher than that of singleton pregnancies. The increased risk associated with twin pregnancies may be related to a combination of multiple factors, such as excessive uterine dilation, hormonal fluctuations, placental abnormalities, and increased maternal burden, thereby increasing the risk of threatened abortion.^26^,^27^ In addition, repeated transplant failures also bring tremendous psychological pressure to pregnant women, thereby increasing the risk of miscarriage.^27^ The levels of beta-hCG, E2, and P on 14 days after IVF-ET are key indicators for evaluating early embryonic development and luteal function in pregnancy.^28^,^29^

In this study, progesterone level was identified as an independent risk factor of threatened abortion. The P level measured 14 days after embryo transfer reflects luteal phase sufficiency and endometrial receptivity, both of which are essential for maintaining pregnancy. Progesterone plays a central role in promoting endometrial transformation into a secretory phase, suppressing uterine contractions, and modulating maternal immune tolerance.^20^ Therefore, a higher serum P level indicates a favorable uterine environment, which significantly lowers the risk of embryo detachment and early miscarriage.^20^ In addition, double embryo transfer, while often increasing pregnancy rates, may also raise the risk of threatened abortion through multiple mechanisms beyond mechanical uterine load. These include abnormal placentation, heightened inflammatory response, suboptimal embryo quality, and excessive hormonal stimulation.^6^,^8^,^14^ Repeated transfer attempts may further reflect previous implantation failures, which could imply latent endometrial receptivity issues or immunologic dysregulation,^8^ both contributing to higher miscarriage susceptibility.

Wang et al.^29^ confirmed that serum beta-hCG levels have a strong independent predictive value for early miscarriage in IVF cycles. E2 and P are crucial for maintaining endometrial receptivity and pregnancy stability.^28^-^30^ E2 promotes endometrial proliferation, while progesterone transforms the endometrium into a secretory phase, creating conditions for embryo implantation and development.^29^,^30^ Notably, only P was included in this nomogram, while beta-hCG and E2 were identified as risk factors. This may be related to the small sample size, as well as to testing stability and statistical factors. The results also indicate that progesterone is most closely associated with threatened abortion after IVF-ET.^30^

Nomograms have been widely used to assess the risk of adverse pregnancy outcomes.^31^ To the best of our knowledge, this is the first nomogram to predict threatened abortion after IVF-ET. The developed model comprehensively evaluates the risk of threatened abortion after IVF-ET by considering multiple factors such as age, endometrial thickness, secondary transplantation, and P on the 14th day of transplantation, rather than relying on single-indicator prediction.^32^ The model has good accuracy in predicting threatened abortion after IVF-ET, with the AUC of 0.822 (95%CI: 0.737-0.907) and 0.822 (95% CI: 0.724-0.919) for the training and validation cohorts, respectively. After calibration, no significant difference was observed between the predicted probability and the observed probability.

Despite favorable AUC and C-index values, the model’s sensitivity was relatively low, particularly in the validation cohort (13.6%), whereas its specificity was consistently high (around 98%). This pattern suggests that the model is more effective in correctly identifying low-risk patients than in detecting all true high-risk cases. Clinically, this implies that the nomogram may serve more appropriately as a confirmation tool to support clinical suspicion in select high-risk IVF-ET populations, rather than as a universal screening tool. This trade-off reflects a deliberate modeling strategy that prioritized minimizing overfitting and improving positive predictive value in a relatively small and imbalanced dataset.

Identifying populations in decision curve analysis has clear clinical significance. The developed nomogram can accurately identify high-risk populations for threatened abortion after IVF-ET, allowing clinicians to take timely preventive measures. In practical terms, the nomogram may be embedded into electronic medical record (EMR) systems to allow automatic risk calculation based on patients’ IVF-ET parameters. This would enable the timely identification of high-risk individuals and facilitate early clinical decision-making. Based on the distribution of scores in our study population and analysis using the Youden index, we suggest a preliminary cutoff score of ≥145, which corresponds to an estimated ≥65% probability of threatened abortion. This cutoff may serve as a reference point for targeted intervention, although further prospective validation is needed.

Patients classified as high-risk by the nomogram may benefit from enhanced early pregnancy monitoring, including more frequent transvaginal ultrasound examinations to assess gestational development. Additionally, physicians may consider optimizing luteal support therapy, such as increasing progesterone dosage or extending treatment duration. Providing individualized psychological counseling may also be beneficial in mitigating anxiety-related risks. These proposed clinical actions enhance the model’s applicability and support its use as a decision-support tool in IVF-ET practice.

This study has several notable strengths. To our knowledge, it is among the first to establish and validate a nomogram specifically designed to predict the risk of threatened abortion after IVF-ET, offering a novel and clinically accessible tool for individualized risk stratification. The model was constructed using a well-defined and homogeneous patient cohort and applied rigorous statistical techniques, including LASSO regression, multivariate logistic regression, ROC analysis, calibration plots, and decision curve analysis, to ensure robustness and clinical relevance. Future research may focus on expanding the model’s functionality by integrating it into clinical decision-support systems and on validating its use across broader IVF populations to enhance its practical utility.

### Limitations:

First, it is a retrospective, single-center study limited to patients undergoing IVF-ET for tubal factor infertility, which may limit the generalizability of the findings. Second, although we aimed to capture early threatened abortion, patients who progressed to spontaneous abortion before data collection were excluded. As a result, the analysis may underrepresent progressive cases and be biased toward non-progressive forms, potentially affecting the model’s external validity. Third, some potentially influential variables—such as lifestyle habits, genetic predispositions, environmental exposures, luteinizing hormone (LH), thyroid function markers (TSH and FT4), and uterine blood flow parameters—were not routinely collected due to the study’s retrospective design. These may act as unmeasured confounders and should be addressed in future prospective, multicenter studies with standardized data collection protocols. Additionally, patients who experienced natural or induced abortion during early or mid-pregnancy were excluded, which limits the applicability of the model to broader IVF populations. Finally, the model requires further external validation in larger, more diverse cohorts. Future work should also aim to improve model sensitivity and clinical utility by integrating additional biomarkers and advanced modeling techniques such as ensemble learning or machine learning–based classifiers.

## CONCLUSION

This study identified age, endometrial thickness, number of transplanted embryos, and P level 14 days after IVF-ET as independent influencing factors for threatened abortion. The nomogram constructed based on these four influencing factors had high accuracy and can be used as a reliable tool for predicting threatened abortion after IVF-ET. Such a nomogram will enable the timely identification of high-risk patients and promote targeted intervention measures. Future studies should further optimize the model, incorporate additional biomarkers and clinical information, enhance prediction accuracy and generalization, and provide more accurate guidance for clinical intervention.

### Authors’ contributions:

**JY:** Study design, literature search and manuscript writing.

**XZ and LX:** Data collection, data analysis and interpretation. Critical review.

**JY:** Manuscript revision and validation and is responsible for the integrity of the study.

All authors have read and approved the final manuscript.

## References

[1] Braverman AM, Davoudian T, Levin IK, Bocage A, Wodoslawsky S (2024). Depression, anxiety, quality of life, and infertility: a global lens on the last decade of research. Fertil Steril.

[2] Wang L, Zhu Y, Wang T, Xu X, Tang Q, Li J (2022). Feasibility analysis of incorporating infertility into medical insurance in China. Front Endocrinol (Lausanne).

[3] Jiang VS, Bormann CL (2023). Artificial intelligence in the in vitro fertilization laboratory: a review of advancements over the last decade. Fertil Steril.

[4] Yu N, Guo Q, Sun M, Sheng Y, Ma Z, Qin Y (2018). Clinical outcomes of IVF/ICSI-ET after thyroid cancer surgical treatment. J Shandong Univ.

[5] Xu Y, Hu X, Ai KL, Sun ZG, Song JY (2025). Gushen Antai pill for expected normal ovarian responders undergoing IVF-ET (GSATP-FreET): interim analysis of a randomized controlled trial. Contracept Reprod Med.

[6] Romanenko TH, Sulimenko OM, Ovcharenko SO (2021). A Statistical Analysis of Obstetric and Perinatal Complications in Singleton and Multiple Pregnancies Once Assisted Reproductive Technologies Are Used. Wiad Lek.

[7] Kouhkan A, Khamseh ME, Pirjani R, Moini A, Arabipoor A, Maroufizadeh S (2018). Obstetric and perinatal outcomes of singleton pregnancies conceived via assisted reproductive technology complicated by gestational diabetes mellitus: a prospective cohort study. BMC Pregnancy Childbirth.

[8] Wang M, Yang X, Li L, Zhu H, Zhang H, Jiang Y (2022). Incidence and risk factors for early pregnancy loss in women with first pregnancy undergoing in vitro fertilization-embryo transfer. BMC Pregnancy Childbirth.

[9] Tong F, Wang Y, Gao Q, Zhao Y, Zhang X, Li B (2024). The epidemiology of pregnancy loss: global burden, variable risk factors, and predictions. Hum Reprod.

[10] Li P, Zhai J, Liu T, Guo M, Wang Y (2024). Comparison of efficacy of long follicular phase regimen and antagonist regimen on pregnancy outcome of fresh cycle or freeze-thawed cycle embryo transfer. Pak J Med Sci.

[11] Borsi E, Potre O, Ionita I, Samfireag M, Secosan C, Potre C (2024). Risk Factors of Thrombophilia-Related Mutations for Early and Late Pregnancy Loss. Medicina (Kaunas).

[12] Yuan S, Liu J, Larsson SC (2021). Smoking, alcohol and coffee consumption and pregnancy loss: a Mendelian randomization investigation. Fertil Steril.

[13] Sheikh MA, Ali S, Khan A (2024). Uroflowmetry: nomograms in healthy young Pakistani men. J Pak Med Assoc.

[14] Huang Y, Wu Y, Jia X, Jia T, Yang Y (2021). Prognostic value of different factors to predict threatened abortion of in vitro fertilization embryo transfer. Chin J Clin Obste Gynec.

[15] Valladares-Garrido MJ, Failoc-Rojas VE, Ichiro-Peralta C, Astudillo-Rueda D, Silva-Díaz H (2022). Toxoplasma gondii Infection and Threatened Abortion in Women from Northern Peru. Infect Dis Obstet Gynecol 2022.

[16] Schieve LA, Tatham L, Peterson HB, Toner J, Jeng G (2003). Spontaneous abortion among pregnancies conceived using assisted reproductive technology in the United States. Obstet Gynecol.

[17] Ozawa N, Ogawa K, Sasaki A, Mitsui M, Wada S, Sago H (2019). Maternal age, history of miscarriage, and embryonic/fetal size are associated with cytogenetic results of spontaneous early miscarriages. J Assist Reprod Genet.

[18] Zhou Y, Yin S, Sheng Q, Yang J, Liu J, Li H (2023). Association of maternal age with adverse pregnancy outcomes: A prospective multicenter cohort study in China. J Glob Health.

[19] Liu L, Liu B, Wu H, Gan Q, Huang Q, Li M (2025). Optimizing predictive features using machine learning for early miscarriage risk following single vitrified-warmed blastocyst transfer. Front Endocrinol (Lausanne).

[20] Gong Y, Jiang T, Sun Y, Wu GL, Han BW, Shi Y (2024). Can single progesterone concentration predict miscarriage in early pregnant women with threatened miscarriage: a systematic review and meta-analysis. BMC Pregnancy Childbirth.

[21] Sheen JJ, Wright JD, Goffman D, Kern-Goldberger AR, Booker W, Siddiq Z (2018). Maternal age and risk for adverse outcomes. Am J Obstet Gynecol.

[22] Pinheiro RL, Areia AL, Mota Pinto A, Donato H (2019). Advanced Maternal Age: Adverse Outcomes of Pregnancy, A Meta-Analysis. Acta Med Port.

[23] Ding H, Tian L (2018). Relationship between endometrial thickness and pregnancy outcomes based on frozen-thawed embryo transfer cycles. Chin J Obstet Gynec.

[24] Ma J, Zhang L, Gao C, Bai X (2025). Influence of the levels of the serum progesterone, estradiol and â-hCG and the endometrial thickness of pregnant women with threatened abortion during the first trimester of pregnancy on their outcomes. Chin J Fami Plan.

[25] Huang W, Tang J, Wei L, Nong L, Tang N, Wei X (2025). Association of endometrial thickness with live birth rates among women undergoing fresh IVF, FET, and PGT cycles. Front Cell Dev Biol.

[26] Balen AH, MacDougall J, Tan SL (1993). The influence of the number of embryos transferred in 1060 in-vitro fertilization pregnancies on miscarriage rates and pregnancy outcome. Hum Reprod.

[27] Tighe J, Broughton S, Roberts R, Kasaven LS, Cutting R, Bridges E (2025). Effectiveness and safety of consecutive single embryo transfer compared to double embryo transfer: results from the UK HFEA registry. Hum Reprod.

[28] Wang L, Wang L, Yang X, Jin P, Zhang R, Jiang Y (2022). Risk factors related to early pregnancy loss in fresh IVF/ICSI: An analysis of 954 embryo transfer cycles. Medicine (Baltimore).

[29] Wang L, Jiang Y, Shen H, Ma X, Gao M, Jin P (2022). Independent value of serum â-human chorionic gonadotropin in predicting early pregnancy loss risks in IVF/ICSI cycles. Front Immunol.

[30] Li L, Zhang Y, Tan H, Bai Y, Fang F, Faramand A (2020). Effect of progestogen for women with threatened miscarriage: a systematic review and meta-analysis. BJOG.

[31] Portal A, Sunyach C, Loundou A, Lacroix-Paulmye O, Perrin J, Courbiere B (2021). Nomograms for predicting adverse obstetric outcome in IVF pregnancy: A preliminary study. Birth.

[32] Ohly NT, Khoury R (2023). Threatened Periviable Delivery and Abortion: Clinical Considerations. Clin Obstet Gynecol.

